# Amphibious Movement of Microrobots Inspired by Water Spiders

**DOI:** 10.1002/advs.77589

**Published:** 2026-09-06

**Authors:** Xiao Hu, Run Ouyang, Zhuhao Shi, Wanqiong Tao, Zuchao Zhu, Peifeng Lin, Jianzhong Lin, Luca Brandt

**Affiliations:** ^1^ Key Laboratory of Fluid Transmission Technology of Zhejiang Province Zhejiang Sci‐Tech University Hangzhou Zhejiang People's Republic of China; ^2^ Department of Environment Land and Infrastructure Engineering (DIATI) Politecnico di Torino Torino Italy; ^3^ Zhejiang Gongshang University Hangzhou Zhejiang People's Republic of China; ^4^ Hefei General Machinery Research Institute Co., Ltd. Hefei Anhui People's Republic of China; ^5^ Faculty of Mechanical Engineering and & Mechanics Ningbo University Ningbo Zhejiang People's Republic of China; ^6^ Department of Mechanics State Key Laboratory of Fluid Power and Mechatronic Systems Zhejiang University Hangzhou People's Republic of China

**Keywords:** amphibious movement, intelligent transportation, magnetic‐driven microrobots

## Abstract

Magnetic actuation provides an effective means for tetherless control of microrobots in complex environments. Inspired by the morphology and interfacial flotation mechanism of water striders, we design a four‐legged microrobot with small ribs on its feet to mimic the structure of water striders. Through an asymmetric magnetic response between its poles inside four legs, two bio‐inspired motion modes and three extended modes are controlled by the magnetic field during complex amphibious locomotion. The microrobot can generate vortices for locomotion on the air‐water interface like water striders; it also keeps the non‐contact cargo‐capturing and transporting capacity with the Rotate motion mode. When switched to the Swing mode, the microrobot can overcome surface tension and enter the liquid interior. In a liquid environment, the microrobot can keep continuous lateral oscillations like water striders; it breaks flow field symmetry to generate vertical displacement. Besides, the microrobot ultimately demonstrates the ability to cross the air‐liquid interface and execute directional movement in sophisticated multi‐element environments and within the complex gastric wall structure, thus ensuring precise controllability. This biomimetic microrobot offers a highly valuable design concept for microrobots requiring amphibious locomotion capabilities.

## Graphical abstract

Inspired by the water strider, a novel microrobot is designed to maintain amphibious mobility in complex environments. It retains the ability to traverse the air‐liquid interface and perform directional movement within sophisticated, multifaceted environments, including complex gastric wall structures, with precise controllability. This bionic microrobot possesses superior functional characteristics and exhibits significant application prospects and potential value in the biomedical field.

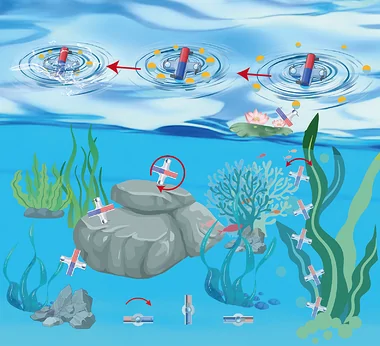

## 1 Introduction

Advances in microfabrication and micro‐nano material technologies enabled the transition of microrobot operations in confined spaces from concept to reality [1, 2, 3]. Swimmers with dimensions ranging from millimeters to micrometers were generally classified as microrobots in previous research [4, 5]. These microrobots demonstrated broad application prospects in numerous fields, such as cargo transport [6, 7, 8], minimally invasive surgery [9, 10, 11], micromanipulation [12], and toxic substance removal [13]. Among various actuation methods, including electric [14, 15], magnetic [16, 17, 18, 19, 20], optical [21, 22, 23], and acoustic fields [24, 25, 26], magnetic actuation was more favored due to its rapid response, wireless controllability, and strong penetration capability.

In recent years, researchers have devoted considerable effort to mimicking various biological functions, aiming to design microrobot structures with novel functionalities and continuously expandable application spaces. Magnetically controlled microrobots inspired by different bionic behaviors, such as inchworms [27], jellyfish [28], sperm [29], cilia [30], beetles [31], tadpoles [32], and sea snake tails [33, 34], have become one of the research hotspots in the field of bionic robotics. Relying on slender limbs for weight distribution and hydrophobic structures for surface flotation, water striders can effectively utilize the surface tension of water to walk on the air‐liquid interface and induce vortex shedding to move rapidly, turn flexibly, and capture prey [35, 36], possessing excellent survival and locomotion capabilities. This also provides new inspiration for the structural design of the microrobot in this paper.

In the field of bionic structural design for microrobots, many researchers have focused on optimizing individual performance characteristics while overlooking the complexity of real‐world operating conditions, which has constrained the applicability of these microrobots. In low‐Reynolds‐number environments dominated by viscous forces, bio‐inspired microrobots with rotating functions could convert rotation into translation to achieve net displacement [37, 38]. However, this motion mode was singular. When encountering a bifurcated pipeline with branches oriented in different directions, the microrobot could only follow a fixed motion pattern, and thus failed to adjust its direction flexibly and accomplish precise drug delivery tasks. Structures inspired by the flagellar motion of sperm or the undulatory locomotion of aquatic organisms enable efficient propulsion in liquids, but microrobots based on these designs typically struggle to achieve three‐dimensional motion when actuated by a single input source [39, 40, 41]. When encountering sharp bends, steep slopes, and trench‐like terrain variations, the microrobot was unable to complete its tasks due to the limited vertical and multi‐directional mobility. Umbrella‐shaped structures inspired by jellyfish and mirror‐symmetric designs modeled after bivalves require ample space for effective displacement [42, 43]. In confined narrow environments, such as tiny human blood vessels or fine branches of industrial pipelines, insufficient space prevented full structural deployment, making it difficult to generate adequate propulsive force for movement. Furthermore, most microrobots were only suitable for liquid environments and lacked amphibious locomotion capabilities [44, 45, 46, 47, 48]. On the gas‐liquid interfaces within biological systems, such as alveolar surfaces in the lungs or in the gastric environment, microrobots could not traverse the air‐liquid interface and failed to meet practical application requirements. In summary, within the field of microrobot research, previously designed structures exhibit issues such as solidified motion modes and oversimplified practical application functions, such as locomotion in complex amphibious environments.

Inspired by the morphology and interfacial flotation mechanism of four‐legged water striders, this paper designs a magnetic field‐driven mimic microrobot with high application potential. The microrobot effectively distributes its weight on four legs to achieve stable suspension on the air‐liquid interface, as the water striders. Furthermore, it mimics the water strider's paddling motion, generating effective displacement by inducing fluid vortex shedding. Through multimodal transition, it achieves efficient motion on the air‐liquid interface, within the liquid, and in complex application scenarios, thereby satisfying amphibious motion requirements. Inside the liquid, magnetic field control enables the microrobot to counteract gravitational forces and complete three‐dimensional motion, effectively resolving fixed‐modality issues in prior microrobots. The designed microrobot demonstrates significant application potential in advanced fields, such as clinical micro‐manipulation and minimally invasive medicine, providing innovative pathways for high maneuverability and complex‐environment operation.

## 2 Results and Discussion

### 2.1 Structure Design and Locomotion Analysis

**FIGURE 1:**
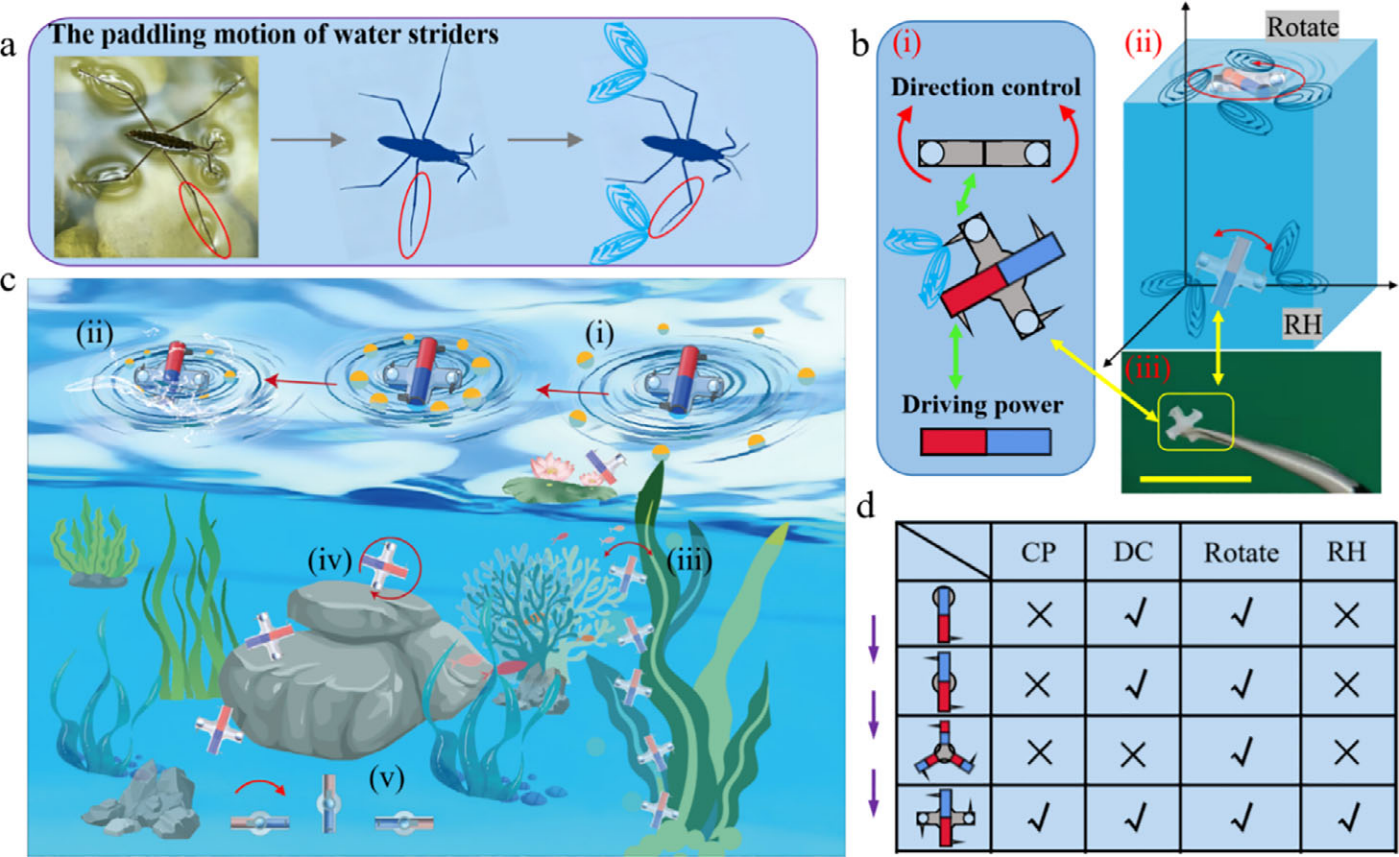
Biomimetic principles and schematic application scenarios of the microrobot. (a) Schematic motion of a water strider on the water surface. (b) Illustrations of the microrobot: (i) schematic structure, (ii) biomimetic Rotate mode and RH mode, (iii) real photo of microrobot, the scale bar is 1 cm, detailed parameters are provided in Figure S3. (c) Application scenario sketches: (i) microrobot captures particles via Rotate mode on the air‐liquid interface, (ii) microrobot enters the liquid interior via Swing mode by beating the surface to overcome interfacial tension, (iii) microrobot climbs the slope via RH mode, (iv) microrobot climbs stairs and (v) walks on the wall by SR mode and Roll mode, respectively. d) Structural optimization process, microrobots with one, two, three, and four legs. A check mark indicates successful function implementation; CP and DC represent capturing particle and directional controlling ability, respectively.

In nature, as shown in Figure 1a, the water strider balances its weight (about 10 mg–80 mg [49]) on four slender legs, and the abundant hydrophobic microstructures on its feet can generate vortices during movement [50], enabling rapid propulsion and locomotion on the water surface. Inspired by the morphology and interfacial flotation mechanism of water striders, as shown in Figure 1a, we design a four‐legged microrobot with a small rib on its feet to mimic the morphology of water striders and achieve flotation. The shell of the microrobot is fabricated via 3D printing using the hydrophobic photosensitive resin (Contact angle tests are shown in Figure S1). By three‐axis magnetic field control (Figure S2), the microrobot features a hollow interior embedding polar magnetic rods (NdFeB magnetic poles) for primary driving force and magnetic beads (Fe_3_O_4_ magnetic beads) for auxiliary actuation, directional offset, and enhanced rotational inertia, thereby producing rotational net displacement (Figure 1b(i)). The microrobot is approximately 6 mg; it is 3 mm wide and 0.5 mm thick (Figure 1b(i)) with ribs on its feet; please refer to Figure S3 for the specific sizes.

By applying a magnetic field ***B***, the magnetic poles generate torque regulated by ***T*** = *V*(***M*** × ***B***) (*V* is the volume of the microrobot, ***M*** represents magnetization), resulting in rigid‐body rotation of the microrobot to align its magnetization with the magnetic field. By altering magnetic field strength (***B***), magnetic field frequency (***f***), and phase difference (***ξ***), this paper identifies five motion modes based on the robot's motion behaviors: (i) Roll, (ii) Stand and roll (SR), (iii) Rise and hover (RH), (iv) Swing, and(v) Rotate modes. (Detailed locomotion behaviors and control details are provided in Figure S4 and Movie S1).

Specifically, through an asymmetric magnetic response between its poles inside the four legs, as shown in Figure 1b(ii),c(i), the microrobot can induce vortices by the Rotate mode on the air‐liquid interface; it also effectively captures and transports particles without contact. By switching to the Swing mode, as shown in Figure 1c(ii), the microrobot beats the liquid surface to break the surface tension, then it enters the liquid interior. Within the liquid environment, as shown in Figure 1b(ii),c(iii), the microrobot keeps the RH mode and can also achieve efficient propulsion in a vertical direction, as the paddling motion of water striders, and overcomes the liquid environment constraints, achieving amphibious applications. Within the liquid environment, the microrobot can also maintain SR mode (Figure 1c(iv)) and Roll mode (Figure 1c(v)) to realise the directional movement near the wall.

As shown in Figure 1d and the specific sizes in Figure S3, the microrobot's structure is continuously optimized by varying the number of legs from one to four. For the tasks of capturing particles (CP), directional control (DC), and realising two biomimetic motions (i. e., Rotate and RH modes), the four‐legged microrobot is identified as the optimal configuration.

### 2.2 Research on the Motion of Microrobots on the Air‐Liquid Interface

Research on the motion of microrobots on the air‐liquid interface holds significant importance, with potential applications in precise drug delivery on the pulmonary air‐liquid interface, detection and removal of floating plastic particles on the water surface, and precise manipulation and assembly of microscale components. This study first investigates the mechanism underlying the capture and transport of plastic particle clusters by a four‐legged microrobot on the air‐water interface.

**FIGURE 2:**
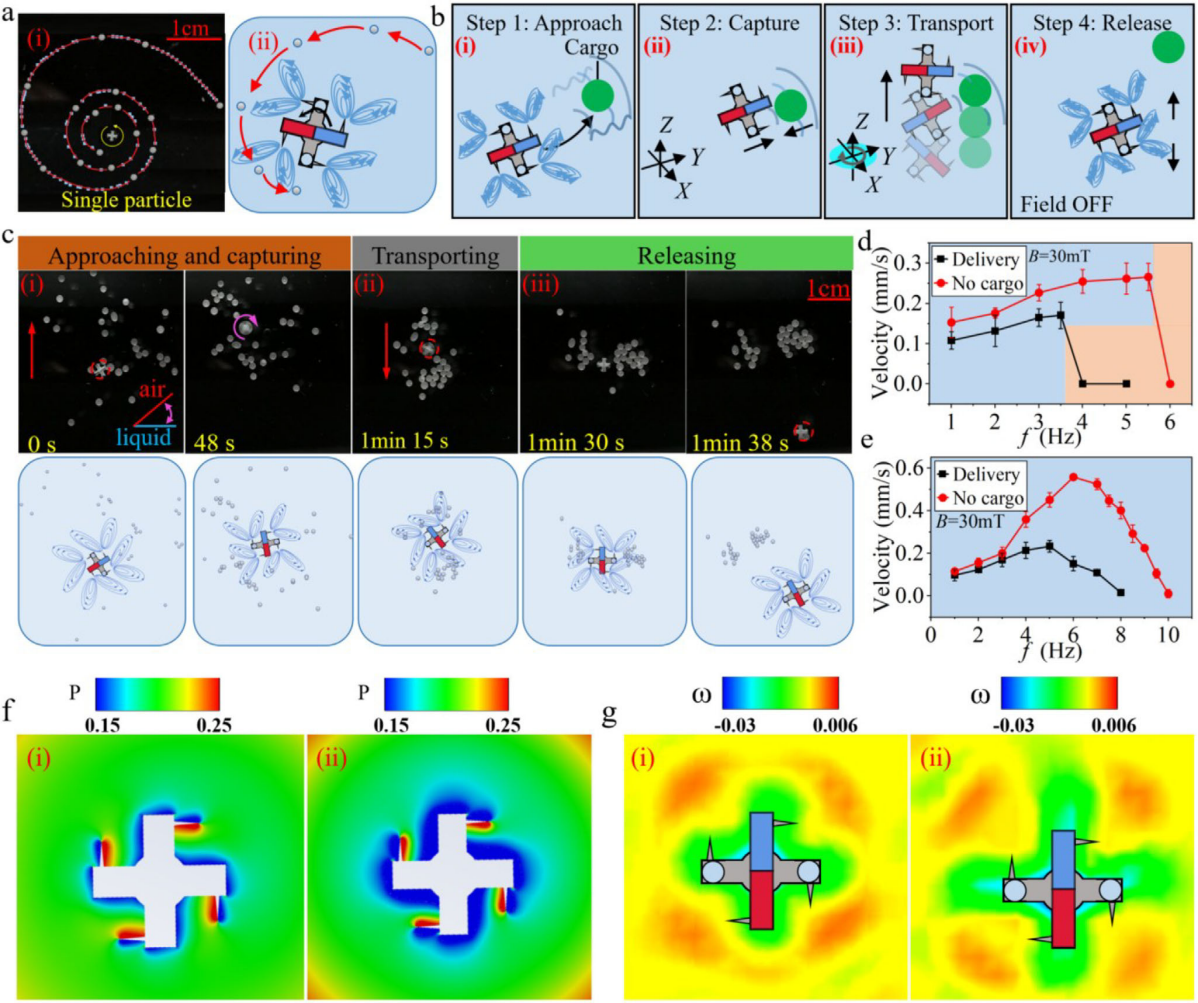
The processes and underlying mechanisms of non‐contact cargo particle capture by the microrobot. (a) Experimental (i) and schematic (ii) views of the motion trajectory of a cargo particle attracted by the rotating microrobot ((*B_x_*, *B_y_*, *B_z_*) = (30mT, 30mT, 0), *f* = 3 Hz). (b) Schematic diagram of the four processes of the microrobot capturing particles. (c) Experimental and schematic images of the process: (i) approach and capture, (ii) transport, and (iii) release of particles. Influence of actuation frequency *f* on the velocity of a microrobot with and without a cargo particle when it keeps (d) the Swing mode or (e) the Rotate mode. (f) Simulated contours of the pressure with *f* = (i) 2 Hz, (ii) 4 Hz when the microrobot rotates on the air‐liquid interface, and (g) the corresponding vorticity contours obtained via PIV when *f* = (i) 2 Hz, (ii) 4 Hz.

Driven by the *X*‐axis and *Y*‐axis magnetic fields, the target particles are attracted close to the microrobot with Rotate mode, as illustrated in Figure 2a. Subsequently, taking cargo particles with diameter *D*_p_ = 1 mm in Figure 2b,c as examples, this study identifies four sequential steps in the particle capture process. First, the magnetic actuation drives the microrobot to keep the Rotate mode, and it approaches the particles (Figure 2b(i)). Second, the microrobot (Figure 2b(ii)) attracts the particles closer and induces co‐rotation (Figure 2a(i‐ii)). Third, the microrobot generates displacement during rotation due to the rotational asymmetry of the magnetic pole (Figure 2b(iii)), and the trapped particles follow the microrobot to achieve directional movement. Lastly, increasing *B*_x_ and *B*_y_ enlarges the rotational amplitude; the microrobot overcomes the liquid bridging effect between itself and the cargo particles (Figure S5), and then the particles are released. The microrobot leaves the release site (Figure 2b(iv)). Figure 2c and Movie S2 illustrate the non‐contact capture and transport of a group of particles by the microrobot, encompassing three stages: approaching and capturing (Figure 2c(i)), transporting (Figure 2c(ii)), and releasing (Figure 2c(iii)) particles.

Subsequently, with (*B_x_*, *B_y_*, *B_z_*) = (30mT, 30mT, 0) and (*B_x_*, *B_y_*, *B_z_*) = (30mT, 0, 30mT), the influence of *f* on the cargo‐carrying velocity is analyzed in Figure 2d,e when the microrobot keeps the Swing and Rotate modes, respectively. The results show that the microrobot exhibits a reduced velocity when carrying particles compared to its unloaded state. When the Swing mode is utilized, it can float and capture particles on the water surface when *f* is small (the blue section). However, the microrobot can beat the liquid surface to break the surface tension and fall to the bottom of the container when *f* is large (orange section in Figure 2d). Maintaining the Rotate mode, the microrobot consistently floats on the air‐water interface, and the motion velocity first increases and then decreases as *f* increases. This phenomenon is attributed to the step‐out frequency. Below the step‐out frequency, the magnetic torque balances the drag torque, and the microrobot moves synchronously; above the step‐out frequency, the magnetic torque fails to balance the drag torque, resulting in a decrease in the motion velocity. Comparing the performance of the microrobot with one, two, three, and four legs in Figure S6, only the four‐legged microrobot successfully captures the particles and achieves directional motion. Consequently, this chapter focuses on the four‐legged microrobot.

To further investigate the mechanism of particle capture, numerical simulation and particle image velocimetry (PIV) experiments are employed when the microrobot rotates on the air‐water interface, and the contours of pressure and vorticity are presented in Figure 2f,g, Figure S7 and Movie S4. During its rotation, the microrobot's ribs disturb the surrounding fluid, accelerating flow velocity and generating a larger velocity gradient, thereby facilitating vortex formation to attract the cargo particles (Figure 2g(i‐ii)); a low‐pressure region forms at the ribs (Figure 2f(i‐ii)). Then the surrounding fluids flow toward this low‐pressure region to replenish it, and the particles are attracted, constituting the mechanism enabling rotational capture and transport of particles by the microrobot.

To verify the hypothesis, we compare the microrobot with and without ribs on its legs in Figure S8 and Movie S3. Under identical operating conditions, the rotation of the microrobot without ribs is insufficient to generate a vortex for particle capture, although particles continue to rotate around it. Additionally, when the sizes of cargo particles are either too small or too large, the microrobot cannot directionally transport particles (refer to Figure S8 and Movie S3).

### 2.3 Locomotion Performance Characterization in Liquid

When the microrobot enters the fluid interior by Swing mode, achieving directional transport to specific destinations with a faster locomotion velocity is equally important. This section further investigates the influence of phase difference (***ξ***), magnetic field frequency (***f***), and magnetic field strength (***B***) on the motion of the microrobot.

**FIGURE 3:**
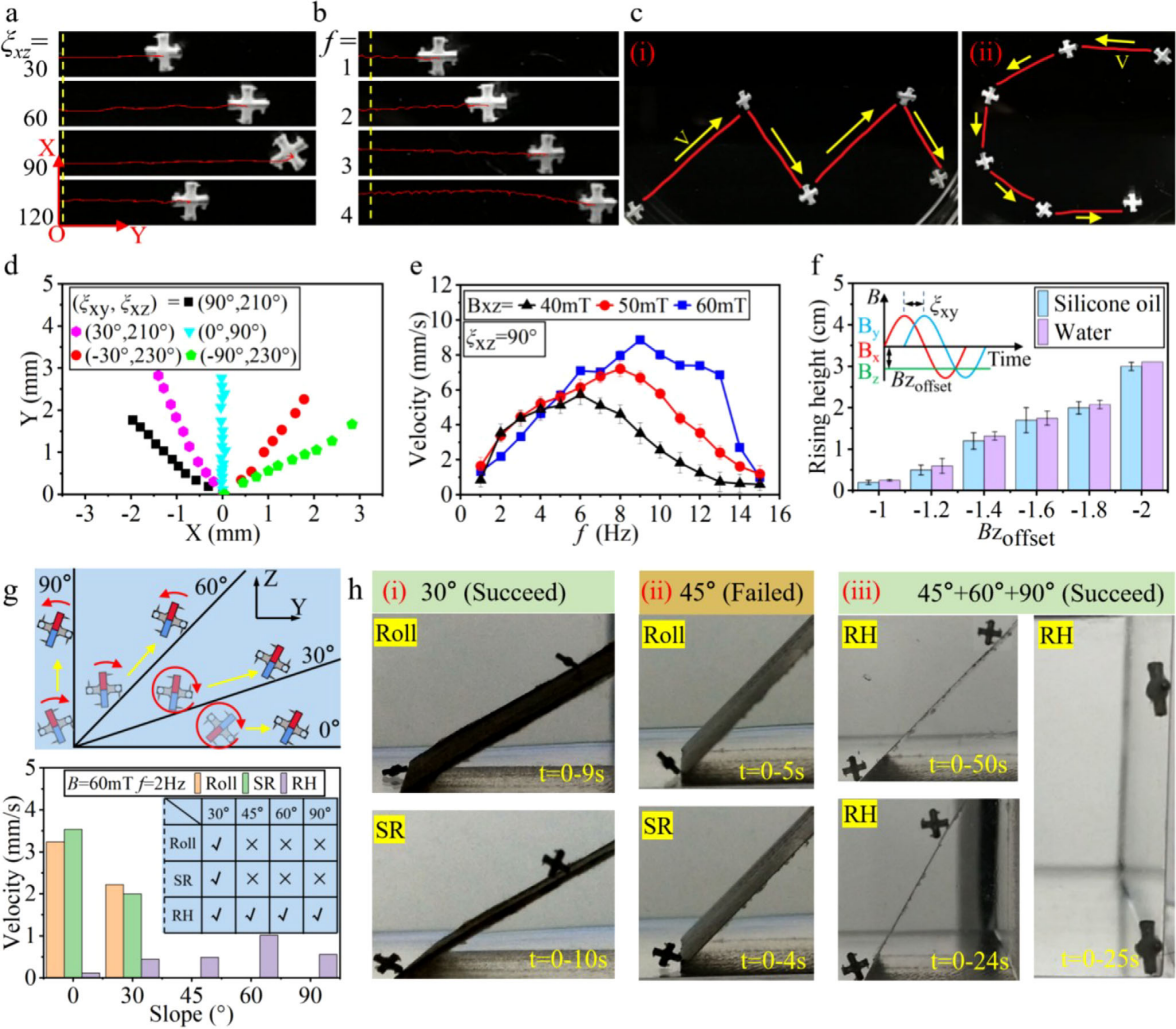
Study on the motion of microrobot inside the liquid. Influence of (a) phase difference ***ξ*** and (b) magnetic field frequency *f* on motion velocity. (c) The microrobot keeps the (i) ‘M’ and (ii) ‘C’‐shaped trajectories. (d) Effect of ***ξ*** on motion direction in water. (e) Effect of *f* on motion velocity under different *B*_xz_ values in water. (f) Variation of vertical ascent height with magnetic bias offset *Bz*_offset_ in silicone oil and water. (g) Locomotion velocities for the microrobot climbing slopes of 30°, 45°, 60°, and 90° by using different motion modes. (h) Experimental images for climbing (i) 30°, (ii) 45°, (iii) 60°, and 90° slopes.

At the same time (*t* = 5 s), the motion trajectory of the microrobot in Figure 3a to b shows that the motion velocity first increases and then decreases as *ξ* increases, while it increases with increasing *f* and ***B*** (please see Movie S5). To achieve directional motion, taking the microrobot's M‐shaped and C‐shaped trajectories as examples, as depicted in Figure 3c and Movie S6, we demonstrate its ability to perform precise directional motion via magnetic control. The effect of ***ξ*** on the motion direction in water is compared in Figure 3d. Adjusting the motion direction depends on the combined action of *ξ*_xy_ and *ξ*_xz_; the motion direction gradually deviates toward the positive *X*‐axis as *ξ*_xy_ decreases and *ξ*_xz_ increases; the microrobot deviates toward the positive *X*‐axis when *ξ*_xy_ increases in the negative direction, and *ξ*_xz_ rises to 230°. For optimal motion velocity, taking (*ξ*_xy_, *ξ*_xz_) = (0°, 90°) as an example, the impacts of ***B*** and *f* on motion velocity in water are analyzed in Figure 3e, where the motion velocity first rises and then falls with increasing *f*. The peak motion velocity is increased as ***B*** rises, which originates from the step‐out frequency of the magnetic poles. Specifically, the microrobot's motion velocity increases with *f* when its magnetic rods track the magnetic field; however, if the rods lag behind the magnetic field frequency, the higher frequencies reduce the velocity.

Actual locomotion scenarios may involve various steep slopes. The microrobot's motion when encountering obstacles is further analyzed. The Roll, SR, and RH modes are selected and tested separately; the corresponding results are shown in Figure 3g,h and Movie S7. At a slope of 30°, microrobots using all three motion modes successfully climb the slope. When the slope increases to 45° and 60°, the Roll and SR modes fail. However, the microrobot can successfully climb the slope by using the RH mode. Previous studies often reported that microrobots were unable to climb slopes steeper than 75° [51, 52, 53], whereas the microrobot proposed in this work even successfully ascends a 90° slope (Figure 3h(iii)). The microrobot's ability to climb the 90° slope on the resin and acrylic materials further confirms that the wall material has minimal impact on climbing velocity (please see Figures S9 and S10). This indicates that the microrobot successfully achieves three‐dimensional spatial motion in highly challenging scenarios like steep slopes, aiding in the expansion of its practical application scope. By employing the RH mode, the microrobot achieves vertical height control ability, and the climbing height is quantified in Figure 3f. As *Bz*_offset_ (the offset of the magnetic waveform from the output axis) increases negatively, the floating height gradually rises, reaching a maximum height of 3.1 cm, which is approximately 10 times the microrobot's own length.

After verifying the efficient motion of microrobots in water, silicone oil is further introduced to conduct a comparative study with water. The rationale behind this choice is twofold: the excellent optical transparency of silicone oil allows for real‐time observation, and its high viscosity closely mimics human body fluids, which can significantly magnify the effect of viscous drag on propulsive efficiency. As compared in Figure S11 and Figure 3f, the effects of ***ξ*** and ***B*** on the motion direction and velocity, and the climbing height of a 90° slope in water and silicone oil are similar. This demonstrates that the magnetic controlling system can stably govern the locomotion of the microrobot across fluid environments with varying viscosities. Experimental tests in Figure S12 indicate that the three‐legged microrobot is incapable of directional motion. Besides, as shown in Figure S13, the four‐legged microrobot maintains a higher locomotion velocity than the other three tested structures even in silicone oil with high viscosity. Thus, this chapter further confirms that the four‐legged microrobot is the optimal structure in a three‐dimensional environment.

### 2.4 Locomotion Performance Characterization in Complex Amphibious Environment

**FIGURE 4:**
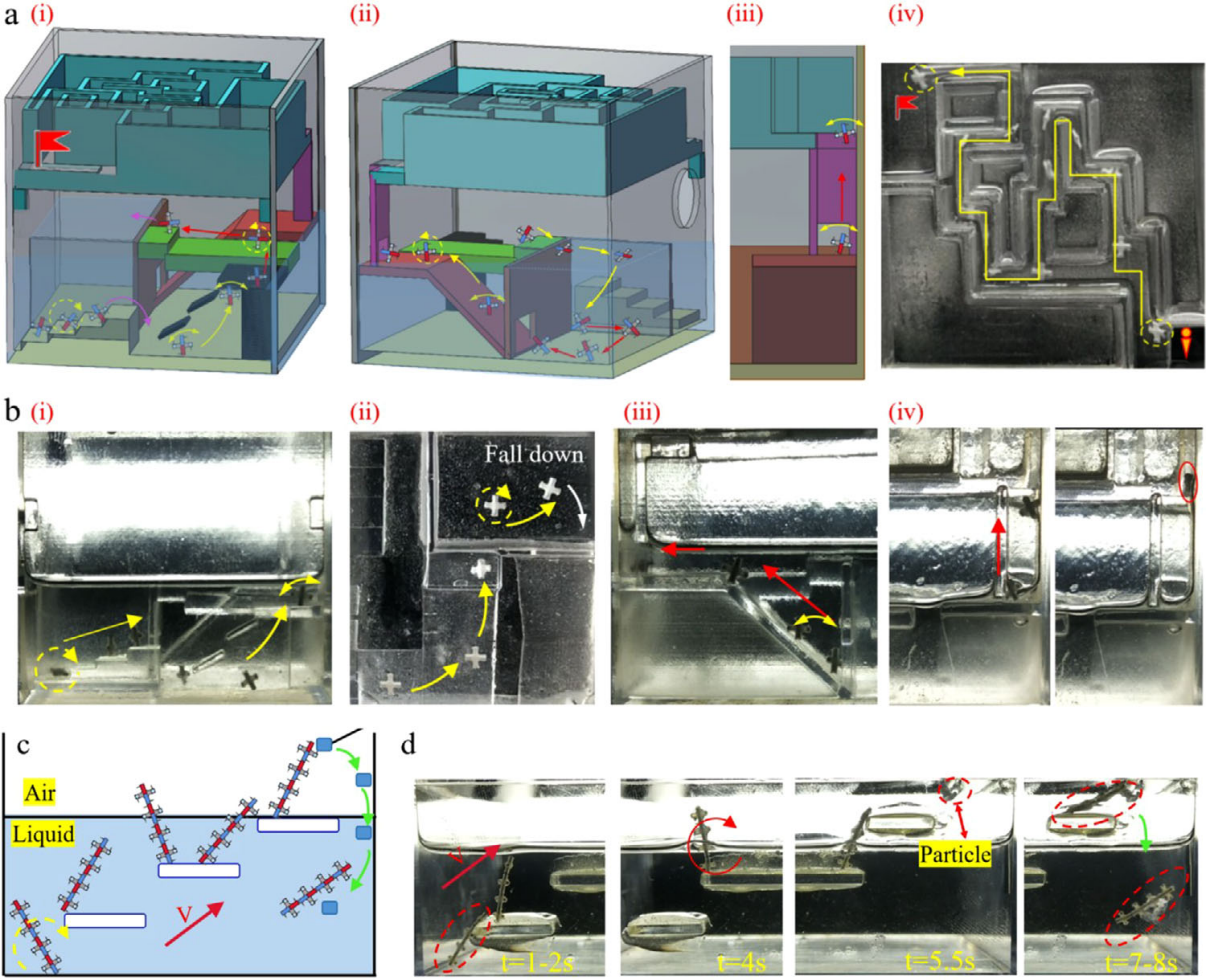
Locomotion of the microrobot in a complex amphibious environment. (a) Schematic of the integrated application scenario; the functions associated with each color are described in Figure S14. (b) Experimental motion behavior in the comprehensive scenario. (c) Schematic and (d) experimental images of the cooperative motion by multiple microrobots, showing transition from a liquid to a gaseous environment and knocking down of a target particle.

A complex amphibious environment is constructed in this study to evaluate the microrobot's maneuverability. The working fluid adopted in this section is water. It integrates stairs, ramps, an aerial platform, a water tank, a 45° slope, a slit with a 90° slope, and a maze (Figure 4a,b). The destination is positioned at the maze's exit (indicated by a red flag). Details of the environment are provided in Figures S14 and S15, and the amphibious locomotion is shown in Movie S8.

After completing the stair‐climbing task by the Roll mode (yellow area, Figure 4a(i)), the microrobot cannot cross a gap wider than its body length by the same mode; it falls to the bottom wall. Then it climbs forward by using RH mode and effectively overcomes gravity and the inclined surface (black area, Figure 4a(i)). When the microrobot reaches the vicinity of the air‐water interface, it climbs a step and enters the air space of the turning platform (green area, Figure 4a(i)) by the Roll mode. After entering the water pool, it keeps rotating and achieves directional motion on the air‐water interface (brown area, Figure 4a(ii)). Subsequently, switching back to the Swing mode, the microrobot utilizes its sharp ribs to rupture the surface tension, sinking into the water and accessing the liquid interior (yellow dashed circle in Figure 4b(ii)). Then the microrobot begins climbing a 45° slope from the door at the pool bottom, completing the seamless connection of amphibious motion (red area, Figure 4a(ii)). When the microrobot fully enters the air, the loss of buoyancy makes its manipulation more challenging compared to the operation in liquid. To reach the destination in the maze, the microrobot must overcome both surface tension in the slit and gravity in air. Utilizing the micro‐ribs and four legs in RH mode, it navigates upward through a 1.6 cm tall, 90° narrow vertical slit (purple, Figure 4a(iii)), which is approximately five times its own length, to access the maze at the second floor. Upon reaching the maze, as shown in Figure 4a(iv), the microrobot cannot navigate the progressively narrowing passages using only single‐motion mode; it must switch between Roll and SR modes to adapt to the changing geometry width. By performing turns and directional movements, the microrobot successfully traverses the maze and arrives at the destination. (Dimensions of the maze are detailed in Figure S15).

Due to the weak buoyancy in air, a single microrobot cannot contact a target particle if its location in air is very high. This limitation can be overcome by employing a combination of four microrobots to achieve collaborative motion, as demonstrated in Figure 4c,d. The target particle exceeds the liquid surface by about 3.1 cm, which is about ten times the height of a microrobot. Maintaining Roll mode enables the microrobots to successfully cross the air‐liquid interface while simultaneously dislodging the target particle into the water (Movie S9). This not only overcomes the constraints imposed by gravity and height difference, but also demonstrates the potential of microrobots for coordinated cooperative motion.

**FIGURE 5:**
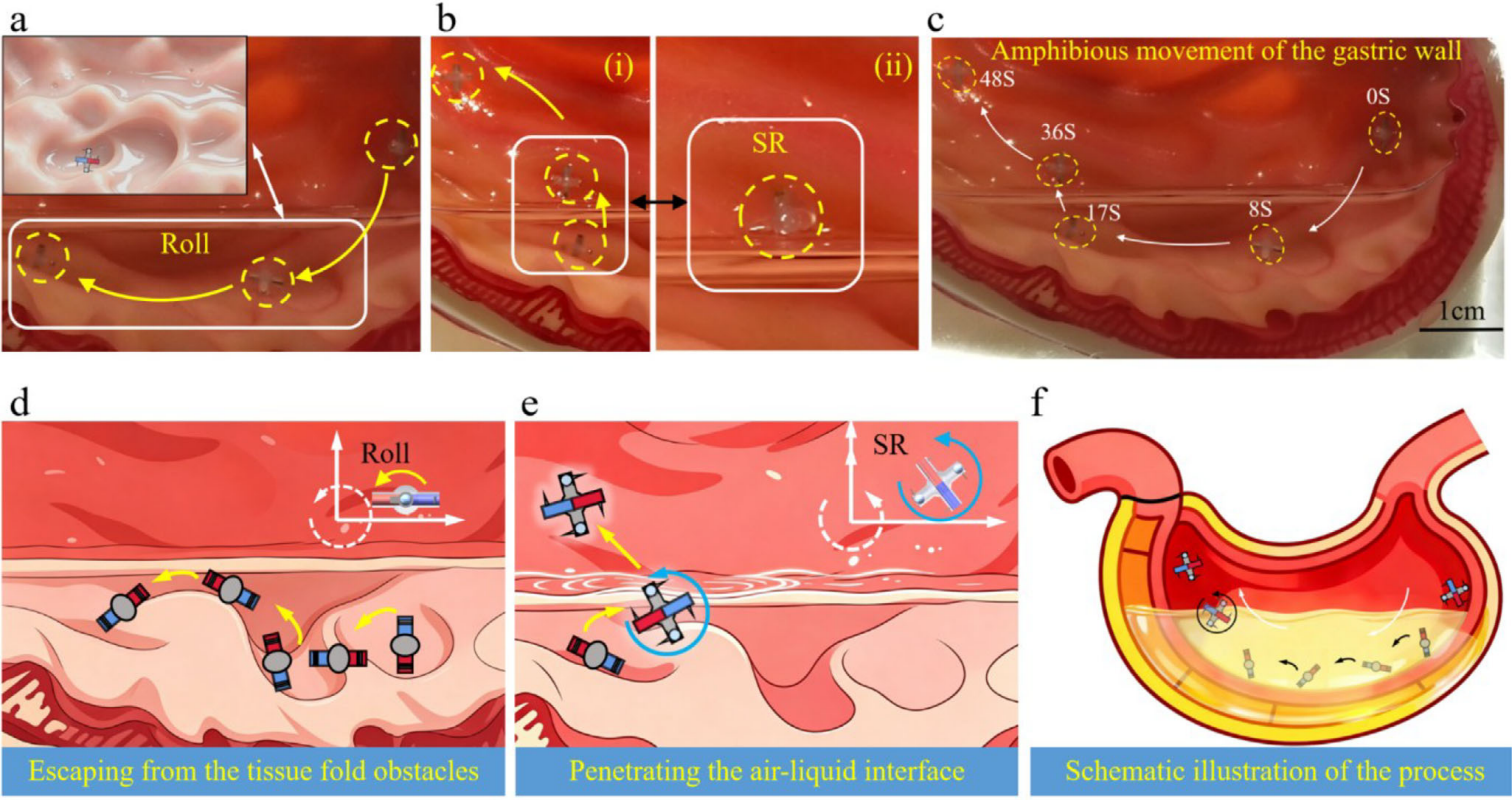
Experimental process of (a) the microrobot reaches the liquid environment and successfully escapes from the tissue fold obstacles, (b) the microrobot penetrates the air‐liquid interface by the SR mode, (c) complete amphibious locomotion in stomach tissue. The corresponding schematic images of (d) escaping from the tissue fold obstacles, (e) penetrating the air‐liquid interface, and (f) complete mode switching process.

As shown in Figure 5 and Movie S10, the microrobot can also perform directional motion within the mucus‐filled folds of stomach tissue (Details of the stomach model can be seen in Figure S16). An epoxy resin model simulating the gastric cavity is used, featuring internal folds with height variations between 2 and 4 mm. These folds act as obstacle zones, which serve to validate the microrobot's capability to overcome adverse topographic conditions. The viscosity of the silicone oil is 100 mPa·s to mimic the typical viscosity range of gastric fluid. Starting from the air on the right wall, the microrobot enters the mucus‐filled stomach model (Figure 5a,d). When the microrobot is trapped in a fold during movement along the stomach wall surface, it can escape from the bottom of the fold by using the Roll mode or SR mode. However, the microrobot cannot penetrate the air‐liquid interface by the Roll mode; it breaches this barrier and reaches the left stomach wall after transitioning to SR mode (Figure 5b,e). Then it accomplishes a vertical ascent of 2.4 cm in air (Figure 5c), demonstrating its amphibious locomotion capability for steep‐slope climbing on folded surfaces. The complete amphibious locomotion process of the microrobot is illustrated in Figure 5f.

## 3 Conclusions

This study proposes a novel magnetically driven bio‐inspired microrobot, which draws inspiration from the morphological structure and interfacial flotation mechanism of water striders. The shell of the four‐legged microrobot is fabricated via 3D printing using the hydrophobic resin to effectively distribute its self‐weight and achieve flotation. The microrobot incorporates an embedded neodymium magnet rod and two magnetic beads. The rod provides the primary driving force, while the beads assist actuation, supply directional deviation, and enhance rotational inertia. Through an asymmetric magnetic response between its poles inside four legs, two bio‐inspired motions (Rotate and RH) modes and three extended (Roll, SR, and Swing) modes are observed during complex amphibious locomotion. The microrobot can generate vortices for locomotion on the air‐ water interface like water striders; it also realizes the non‐contact transport of cargo particles by the rotate mode. Furthermore, by precisely regulating its motion modes, the microrobot ultimately demonstrates the ability to cross the air‐liquid interface and execute directional movement in sophisticated multi‐element and amphibious environments (such as mazes, steps, slopes, and water pools). Subsequently, an amphibious scenario is constructed, designed to mimic the surface features of the stomach. Experimental results demonstrate that the microrobot can traverse various complex biological obstacles. These include the viscous resistance of mucus and path disruptions caused by tissue folds, highlighting its robust obstacle‐crossing adaptability. The designed microrobot is suitable for fields such as early disease diagnosis, targeted drug delivery, and minimally invasive surgery. It possesses superior functional characteristics and exhibits extremely broad application prospects and potential value in the biomedical field.

## 4 Experimental Section

### 4.1 Materials

A neodymium‐iron‐boron (NdFeB) magnetic rod (MQFP‐B‐F00383, average length: 3 mm) is purchased from Magnequench, with a density of approximately 7.4 g/cm^3^. Fe_3_O_4_ magnetic beads (average size: 0.5 mm) are obtained from Feihong Magnet, exhibiting a density of about 4.5 g/cm^3^. The microrobot shell, fabricated from high‐precision photosensitive Acrylonitrile‐butadiene‐styrene (ABS) resin (density: 1.04 g/cm^3^), is sourced from Maer Software Technology Development Co., Ltd. The total mass of the microrobot is approximately 6 mg, resulting in an overall density of about 1.39 g/cm^3^. Furthermore, the contact angle of the ABS resin in Figure S1 indicates that it is an oleophilic and hydrophobic material. Polystyrene (PS) microsphere particles with varying diameters (*D*_p_ = 0.5 mm, 1.0 mm, and 4.0 mm; density is 1.05 g/cm^3^) are purchased from Hongyu Plastic Co., Ltd.

### 4.2 Magnetic Characterization

Magnetic fields are generated using three‐dimensional Helmholtz coils, which generate a magnetic field within a workspace of 50 mm × 55 mm × 55 mm. A Gauss meter (MG801, Magna, Japan) measures the magnetic flux density at a fixed point and direction. A magneto‐optical sensor (MagView‐S, Matesy, Germany) characterizes the distribution of surface magnetic flux density. Magnetic fields are produced by custom‐made Helmholtz coils connected to a programmable power supply. The magnetic field parameters, including waveform, strength, frequency, and bias, are configured via a signal generator. The magnetic field signals are calibrated using an oscilloscope. Additionally, the switching between continuous motion modes is controlled by LabVIEW.

### 4.3 Solution

Distilled water is adopted in the work, whose density is 0.9982 g/cm^3^ at 20°C. Two viscosity grades of silicone oil with 50 mPa·s and 100 mPa·s and a purity of ≥ 99% are used in the experiments; they are purchased from the Creative Dow Corning Silicone Oil Store. The densities of the silicone oils at 20°C are 0.950 g/cm^3^ (50 mPa·s) and 0.960 g/cm^3^ (100 mPa·s).

### 4.4 Video Recording

All macro videos and images are captured using a camera (D7500, Nikon, Japan) equipped with a macro lens (Sigma, 105 mm/F2.8). Data extraction from images or videos is based on ImageJ, in‐house Python code, and Tracker. First, spatial calibration is performed to convert pixel‐based coordinates into physical units. Subsequently, the trajectory of the microrobot is extracted by tracking the centroid of the object across consecutive frames. The instantaneous velocity is calculated by dividing the frame‐to‐frame displacement by the time interval (derived from the acquisition frame rate). All experiments are repeated three times.

## Author Contributions

**Xiao Hu**: conceptualization, formal analysis, supervision, resources, project administration, funding acquisition, writing – review and editing. **Run Ouyang**: software, data curation, validation, investigation, visualization, writing – original draft. **Zhuhao Shi**: validation, software, visualization, investigation. **Wanqiong Tao**: project administration, formal analysis, software, funding acquisition. **Zuchao Zhu**: software, project administration, funding acquisition. **Peifeng Lin**: project administration, funding acquisition. **Jianzhong Lin**: conceptualization, funding acquisition, project administration. **Luca Brandt**: conceptualization, project administration, resources, supervision, software. All authors contributed equally.

## Conflicts of Interest

The authors declare no conflicts of interest.

## Supporting information




**Supporting File 1**: advs77589‐sup‐0001‐SuppMat.doc.

**Supporting File 2**: advs77589‐sup‐0002‐MovieS1.mp4.

**Supporting File 3**: advs77589‐sup‐0003‐MovieS2.mp4.

**Supporting File 4**: advs77589‐sup‐0004‐MovieS3.mp4.

**Supporting File 5**: advs77589‐sup‐0005‐MovieS4.mp4.

**Supporting File 6**: advs77589‐sup‐0006‐MovieS5.mp4.

**Supporting File 7**: advs77589‐sup‐0007‐MovieS6.mp4.

**Supporting File 8**: advs77589‐sup‐0008‐MovieS7.mp4.

**Supporting File 9**: advs77589‐sup‐0009‐MovieS8.mp4.

**Supporting File 10**: advs77589‐sup‐0010‐MovieS9.mp4.

**Supporting File 11**: advs77589‐sup‐0011‐MovieS10.mp4.

## Data Availability

The data that supports the findings of this study are available in the supplementary material of this article.
