# Supplementary material for: Amphibious Movement of Microrobots Inspired by Water Spiders
Source: Adv Sci (Weinh). 2026 Sep 6:e77589. Online ahead of print. doi: 10.1002/advs.77589 (PMC13547543; doi:10.1002/advs.77589)
Supplement: Supplementary file 1 [file ADVS-9999-e77589-s003.docx]

Supporting Information

**Amphibious Movement of MicroRobots Inspired by Water spiders**

*Xiao Hu*^1, 6[1]^, *Run Ouyang*^1^, *Zhuhao Shi*^1^, *Wanqiong Tao*^2^, *Zuchao Zhu*^1, 3[2]^, *Peifeng Lin*^1^, *Jianzhong Lin*^4, 5^,

*Luca Brandt*^6^

*^1^ Key Laboratory of Fluid Transmission Technology of Zhejiang Province, Zhejiang Sci-Tech University, Hangzhou, Zhejiang 310018, PR China*

*^2^ Zhejiang Gongshang University, Hangzhou, Zhejiang 310027, PR China.*

*^3^ Hefei General Machinery Research Institute Co., Ltd., Hefei, Anhui 230031, PR China*

*^4^ Faculty of Mechanical Engineering and & Mechanics, Ningbo University, Ningbo, Zhejiang 315201, PR China*

*^5^ Department of Mechanics, State Key Laboratory of Fluid Power and Mechatronic Systems, Zhejiang University, Hangzhou 310027, China*

*^6^ Department of Environment, Land and Infrastructure Engineering (DIATI), Politecnico di Torino, Corso Duca degli Abruzzi 24, Torino, 10129, Italy*

**This PDF file includes:**

Supplementary text

Supplementary Figure 1 to 15.

Legends for Supplementary Movie S1 to Movie S10.

**Supplementary Note 1. Test of contact angle**

Contact angle measurement is performed on the same material used for the microrobot shell (Acrylonitrile-butadiene-styrene (ABS) resin), the results are presented in Figure S1. The contact angles of the resin wall surface measured with water and silicone oil are 98.3° and 27.5°, respectively. The contact angle reveals that the material is oleophilic and hydrophobic. This property ensures that, even though the effective density of the microrobot exceeds that of water, the gravity can be balanced by surface tension and buoyancy, thus ensuring the stable flotation of the microrobot.

**Figure S1.** The measurement of the contact angles of the resin wall surface by (a) water and (b) silicone oil.

**Supplementary Note 2. The magnetic field control mechanism of magnetic drive.**

To achieve precise controlling of microrobots, the magnetic field induction strength of a rotating magnetic field is used:

$B\left(t\right)=B_{0}\left(\sin\left(2\pi ft\right),\sin\left(2\pi ft-\xi_{xy}\right),\sin\left(2\pi ft-\xi_{xz}\right)\right)$ (1)

where *t* [s] is the time, *f* [H_Z_] is the frequency of magnetic field, *ξ*_xy_ and *ξ*_xz_ are the phase difference in the *X*o*Y* and *X*o*Z* planes. The magnetic induction intensity ***B***_0_ is:

$B_{0}=\frac{\mu I\left(t\right)N}{\sqrt{L^{2}+\left(4R^{2}\right)}}$ (2)

where *μ* [H/m] is the magnetic permeability of free space, ***I****(t)* [A] is the current through the coil, *N* is the number of turns in the coil, *R* [m] is the radius of coil.

When other parameters are kept the same value, the magnetic field strength within the coil is only impacted by the current magnitude. The real-time current magnitude formula ***I***(*t*) is:

$I(t)=I_{0} sin(\omega t+\xi)$ (3)

where ***I***_0_ [A] is the current amplitude, *ω* [rad/s] is the angular frequency, *ξ* [°] is the phase difference.

**Figure S2.** Schematic diagram of the magnetic field control process.

The system is divided into three parts:

(1) The observation part: there are cameras in the *Z*- axis and *X*- axis directions, which are responsible for capturing the actual movement of the microrobot.

(2) The control part: the control part consists of a computer, an oscilloscope, and a power amplifier. Among them, the oscilloscope controls the magnetic field information, such as magnetic field strength, magnetic field frequency, and waveform.

(3) The experiment part: there is a three - dimensional Helmholtz magnetic coils, which generates a magnetic field within a workspace of 50 mm × 55 mm × 55 mm.

**Supplementary Note 3. Schematic diagram and physical picture of the microrobot and its sizes.**

Here are the structures of four types of micro-robots that have been designed. The shell of the microrobot is fabricated via 3D printing using the hydrophobic photosensitive resi. The microrobot features a hollow interior embedding polar magnetic rods (NdFeB magnetic poles). magnetic beads (Fe_3_O_4_ magnetic beads) are introduced into the four-legged microrobot. Through continuous iterative optimization, the models have evolved as one- two-, three- and four-legged microrobots, the corresponding sizes can be seen in Figure S3.

**Figure S3.** Schematic diagram and physical picture of the structural iteration parameters of the microrobot. a) One-legged micro-robot; b) Two-legged micro-robot; c) Three-legged micro-robot; d) Four-legged micro-robot.

**Supplementary Note 4. Division of motion modes and schematic diagram of the magnetic field.**

The motion modes found in this study include the Roll mode, Stand and roll (SR) mode, Rise and hover (RH) mode, Swing mode, and Rotate mode. The motion characteristics and parameter tuning ranges for each mode are detailed as follows:

(a) Roll mode: The motion behavior is characterized by the flattened microrobot performing a full forward roll. The experimental parameter ranges utilized are: magnetic field strength (*B*_x_, *B*_y_, *B*_z_) = (20~60mT, 0, 20~60mT), magnetic field frequency (*f*_x_, *f*_y_, *f*_z_) = (1~15Hz, 0, 1~15Hz), *ξ*_xz_=90°.

(b) Stand and roll mode: The motion behavior is characterized by the standing microrobot performing a full lateral roll. The experimental parameter ranges utilized are: magnetic field strength (*B*_x_, *B*_y_, *B*_z_)=(60mT, 0, 60mT), magnetic field frequency (*f*_x_, *f*_y_, *f*_z_)= (1~4Hz, 0, 1~4Hz), *ξ*_xz_=90°, *B*_zoffset_=-0.4.

(c) Rise and hover mode: The motion behavior is characterized by periodic vertical oscillations (lifting and descending), wherein the height of the rise and fall is modulated by the *B*_zoffset_. The experimental parameter ranges utilized are: magnetic field strength (*B*_x_, *B*_y_, *B*_z_)=(0~20mT, 0~20mT, 0~200mT), magnetic field frequency (*f*_x_, *f*_y_, *f*_z_)= (1~4Hz, 1~4Hz, 0), *ξ*_xz_= (50°~150°), *B*_zoffset_ = (-3.0~0).

(d) Swing mode: The motion behavior is characterized by a periodic cycle in which the microrobot repeatedly lifts and lowers one of its ends. The experimental parameter ranges utilized are: magnetic field strength (*B*_x_, *B*_y_, *B*_z_) = (0~30mT, 0, 0~30mT), magnetic field frequency (*f*_x_, *f*_y_, *f*_z_) = (1~6Hz, 0, 1~6Hz), *ξ*_xz_=30°.

(e) Rotate mode: The motion behavior is characterized by the flattened microrobot performing a rotation. The experimental parameter ranges utilized are: magnetic field strength (*B*_x_, *B*_y_, *B*_z_)=(20~60mT, 0, 0), magnetic field frequency (*f*_x_, *f*_y_, *f*_z_)= (1~13Hz, 0, 1~13Hz), *ξ*_xy_=90°.

All the aforementioned motion modes are configured using a signal generator and calibrated via an oscilloscope. For experimental sequences requiring continuous mode switching, the operation is pre-programmed using LabVIEW.

**Figure S4.** Modal division: (a) Roll mode, (b) Stand and roll mode, (c) Rise and hover mode, (d) Swing mode, (e) Rotate mode.

**Supplementary Note 5. Schematic diagram of the four-legged microrobots attracting particles via Rotate mode.**

Surface tension is the pulling force between adjacent parts of liquid surface. It enables the liquid surface to shrink as much as possible to reach a stable state with the lowest energy. On the liquid surface, the internal attractive force exerted on water molecules is greater than the attractive force of external air molecules, resulting in a resultant force directed towards the interior of the liquid. This causes the liquid surface to have a tendency to contract.

When the microrobot rotates on the air-liquid interface, as shown in Figure S5(a-b), it will be slightly immersed in a liquid to achieve a stable state. The liquid, in order to reduce its surface energy, forms a continuous liquid connection between the two solid surfaces, which is called a liquid bridge. The small particle will be attracted by the robot as two solid surfaces come close (Figure S5(c)). The liquid bridge force can serve as an effective manipulation force for grabbing and moving tiny particles or devices.

**Figure S5.** a) Three-dimensional schematic of the rotating microrobot on the air-liquid interface. b) A schematic diagram showing the robot slightly immersed in the liquid surface. c) Schematic diagram of the microrobot floating on the liquid surface with Rotate mode and capturing particles.

**Supplementary Note 6. Comparison of the particle-capturing capabilities of four types of robots on the air-water interface.**

As shown in Figure S6, the robot's structure is continuously optimized by varying the number of legs from one to four. A check mark indicates successful function implementation. AC, TR, and RE denote approaching and capturing, transporting, releasing process, respectively. Only the four-legged model can realize the process of approaching and capturing, transporting, releasing particle, which is identified as the optimal configuration

**Figure S6.** Comparison of the particle-capturing capabilities of four types of robots on the air-liquid interface.

**Supplementary Note 7. Computational fluid dynamics simulations**

To further investigate the particle capture mechanism at the air-liquid interface, CFD simulations are conducted using the commercial software ANSYS Fluent (ANSYS, Inc., USA). Specifically, the underlying mechanism is analyzed by comparing the pressure contours generated during the microrobot's rotation.

After the geometric modeling is completed, the generated mesh is presented in Figure S7. The minimum size of the surface mesh is set to 0.00002 m, and the maximum size is set to 0.0001 m. When generating the volume mesh, polyhedra is selected as the filling volume mesh type. The global size control method is adopted, with a growth rate of 1.2 and a maximum mesh length of 0.0001 m.

**Figure S7**. a) Rotational simulation domain. b) the mesh on the microrobots.

The multi - phase VOF model is adopted. Phase 1 is the liquid phase, with a density *ρ* of 998 kg/m³ and a dynamic viscosity *μ* of 0.001 kg/(m·s). Phase 2 is air, and the surface tension is set to 0.072 N/m.

The interface between multiple phases is captured by solving the phase volume fraction transport equation. For the K-th phase, the governing equation is:

$\frac{\partial\alpha_{q}}{\partial t}+\nabla\alpha_{q}=0\left(q=1,2\right)$ (4)

To resolve the additional forces (e.g., Coriolis and centrifugal forces) induced by fluid-structure interaction effects in rotating machinery, the momentum equation in a rotating coordinate system is described by the incompressible Navier-Stokes equations in conservative form:

$\frac{\partial}{\partial t}\left(\rho\overrightarrow{u}\right)+\left(\rho\overrightarrow{u}\cdot u\right)=-\nabla p+\nabla\cdot\left[\mu\left(\nabla\overrightarrow{u}+\nabla\overrightarrow{u^{T}}\right)\right]+\rho\overrightarrow{g}+\overrightarrow{F}_{st}$ (5)

The pressure - based transient solution method is used, and the flow state is set as the laminar flow. The SIMPLE coupling algorithm is adopted, with a time step Δ*t* of 2×10⁻⁴ s and 30 iterations per time step.

**Supplementary Note 8. Details of the PIV experiments**

To further investigate the mechanism of capturing particle, particle image velocimetry (PIV) experiments are employed when the microrobot rotates on the air-water interface, and the contours of vorticity are calculated.

The diameter of the tracer particles ranges from 9 to 13 micrometers. Adjust the laser system to emit high- intensity pulsed laser beams. Use optical elements to shape and expand the laser beam into a laser sheet with a uniform thickness that can fully cover the measurement area. Turn on the magnetic field to drive the microrobot to rotate stably, and synchronously trigger the laser and camera systems. Multiple sets of tracer particle image pairs are continuously acquired at pre-defined time intervals to track particle positions over time. Then the velocity field and vorticity field maps are compared in this article.

On the air-water surface, the velocity field in a two‑dimensional Cartesian coordinate system is given by:

 (6)

where *u*_x_ and *u*_y_ are the velocity components in the *x* and *y* directions, respectively.

Vorticity, which quantifies the local rotation of fluid elements, is defined as the curl of the velocity field:

$\overrightarrow{\omega}=\nabla\times\overrightarrow{u}$ (7)

**Supplementary Note 9. Examples of unsuccessful particle capture.**

We further investigate the capture process of microrobots for particles with *D*_p_ = 0.5 mm and 4 mm. When *D*_p_ = 0.5 mm, as shown in Figure S8(a) and Movie S3, even when the particles are very close to the microrobot (about 0.8 cm), the particles are captured by the micro - vortices around the microrobot, but they cannot get closer. Therefore, the particles are not captured. When the particle size is increased to *D*_p_ = 4.0 mm, as shown in Figure S8(b) and Movie S3, the microrobot is captured by the particle after approaching it, indicating that there is an upper limit for the size of the particles that can be captured.

The ability of the microrobot to capture and transport particles is attributed to the fact that the ribs can cut the fluid more effectively, generating a larger velocity gradient, which makes it easier to trigger vortices to attract particles. To verify this conjecture, a control experiment is conducted, as shown in Figure S8(c) and Movie S3. Under the same working conditions, the rotation of the microrobot without ribs is insufficient to capture particles, although the particles still rotate around it.

**Figure S8.** Examples of unsuccessful particle capture: a) the cargo particle size is too small, *D*_p_ = 0.5 mm. b) the cargo particle size is too large, *D*_p_ = 4 mm. c) Microrobot has no ribs, *D*_p_ =1 mm.

**Supplementary Note 10. The climbing motion of microrobots with different materials.**

The climbing motion behaviour of microrobots on surfaces of the acrylic and resin are compared in Figure S9. For easy observation, a resin block is selected and pasted on the acrylic wall surface. The microrobot adopts the RH mode to perform a climbing motion on both material surfaces.

**Figure S9.** Experimental images of the microrobots climbing on (a) resin and (b) acrylic surfaces.

The quantitative results of climbing velocity in Figure S10 show that the difference in its motion velocity is small.

**Figure S10.** Velocity tests of microrobots climbing on resin and acrylic surfaces.

**Supplementary Note 11. Comparison of motion direction and velocity in water and silicone oil.**

A comparative study is conducted in water and silicone oil to evaluate the microrobot's control over its motion direction and velocity. As compared in Figure S11, the effects of ***ξ*** and ***B*** on the motion direction and velocity in water and silicone oil are similar. This demonstrates that the magnetic controlling system can stably govern the locomotion of the microrobot across fluid environments.

**Figure S11.** Comparison of microrobot (a) motion direction and (b) velocity in water and silicone oil.

**Supplementary Note 12. Comparison of motion direction and velocity among the four robot structures.**

Microrobots need to have precise direction controlling capacity and multiple motion modes to handle complex environments and meet the practical requirements. When the magnetic field is used for directional control, as shown in Figure S12, the three-legged model exhibits significant motion deviation, while the four-legged microrobot can keep the straight trajectory under the same magnetic condition ((*B*_x_, *B*_y_, *B*_z_) = (0, 40mT, 40mT), (*f*_x_, *f*_y_, *f*_z_) = (0, 1Hz, 1), *ξ*_yz_=90°). Thus, the three-legged microrobot cannot meet the requirements for precise directional control.

**Figure S12.** Controlling of motion direction for (a) three-legged and (b) four-legged microrobots.

In order to compare the motion velocity among the four robot structures, the experimental results about the locomotion inside the liquid are presented in Figure S13. Due to the fact that the working fluid environment with high viscosity is critical for microrobot control, silicone oil with viscosity of 50 mPa·s is selected in this experiment. The Roll mode is used, which is driven by the rotating magnetic fields in the *X* and *Z* directions, and *B*_xz_ = 40 mT.

As shown in Figure S13, the velocity difference between the one-legged and two-legged models is not significant. The four-legged microrobot achieves precise directional control and significantly higher locomotion velocity than the others. Thus, the four-legged model can achieve precise direction control and has multiple motion modes to capture particles and adapt to complex amphibious environments, which is identified as the optimal configuration.

**Figure S13.** Changes of motion velocity of four kinds of microrobots.

**Supplementary Note 13. Functional introduction of the complex amphibious environment.**

The integrated scenario includes functions such as climbing stairs (yellow area), climbing the sloped roof (black area), turning in the air (green area), entering the swimming pool, sinking to the bottom, and exiting through the passage door at the pool bottom (brown area), climbing a 45° slope (red area), climbing to the second floor in the slit with 90° slope (purple area), and navigating through a maze.

 **Figure S14.** Schematic diagram of each component on the first layer of the integrated scenario.

**Supplementary Note 14. Dimensional parameters of the maze.**

This is a detailed schematic diagram of the maze dimensions in the complex amphibious environment. The position marked by the red flag in the maze is the destination for microrobot motion.

The specific parameter settings are shown in the right table. The dimensions of the channels width are reduced slowly. Therefore, when using the microrobot to pass through, it is necessary to continuously switch the motion modes.

|  | channel | width (mm) |
| --- | --- | --- |
|  | a | 5.87 |
|  | b | 2.91 |
|  | c | 2.53 |
|  | d | 3.21 |
|  | e | 2.31 |

**Figure S15.** The specific dimensions of the maze.

**Supplementary Note 15. A gastric cross-section model made of epoxy resin.**

Here are the dimensions and actual pictures of the stomach model. The surface of the stomach has a large number of folds, an amphibious environment is formed when there is liquid inside the model. The microrobot can move in this amphibious environment, which proves the superiority of the microrobot.

**Figure S16.** Stomach models and their length and width dimension parameters.

Supplementary Movie S1 - Modal division.

Supplementary Movie S2 - Multi-particle capture, transportation and release.

Supplementary Movie S3 - Single particle capture.

Supplementary Movie S4 - Vorticity contour map obtained via PIV.

Supplementary Movie S5 - Motion states under different conditions of *ξ*_xz_, *f* and ***B****.*

Supplementary Movie S6 - Four-legged microrobot achieves the M- and C-shaped trajectories.

Supplementary Movie S7 - Climb slopes from 30° to 90° by different modes.

Supplementary Movie S8 - Comprehensive movements in complex scenarios.

Supplementary Movie S9 - Microrobots collaboratively dislodge airborne suspended particles.

Supplementary Movie S10 - Amphibious movement within stomach tissue.

1. Corresponding author. *E-mail address*: [huxiao0329@163.com](mailto:huxiao0329@163.com)
2. Corresponding author. *E-mail address*: [zhuzuchao@hrmri.com](mailto:zhuzuchao@hrmri.com)
